# Shrinkage Reduction in Nanopore-Rich Cement Paste Based on Facile Organic Modification of Montmorillonite

**DOI:** 10.3390/ma17040922

**Published:** 2024-02-17

**Authors:** Fengyuan Yang, Ying Yang, Shaoyou Chen, Chao Jin, Jun Jiang, Tie Liu, Fei Lv, Chenxi Yang, Zhongyuan Lu, Jun Li

**Affiliations:** 1 State Key Laboratory of Environmentally Friendly Energy Materials, School of Materials and Chemistry, Southwest University of Science and Technology, Mianyang 621010, China; horizonyfy@139.com (F.Y.); yangyingckx@163.com (Y.Y.);; 2 Ningbo Construction Engineering Group Co., Ltd., Ningbo 345040, China; chensy202401@163.com (S.C.); jinchaodennis@163.com (C.J.);

**Keywords:** nanopore-rich cement paste, shrinkage, pore structure, organic modification, montmorillonite

## Abstract

The organic modification of montmorillonite was successfully achieved using cetyltrimethyl ammonium bromide under facile conditions. The modified montmorillonite was subsequently used for the fabrication of montmorillonite-induced nanopore-rich cement paste (MNCP), and the shrinkage behavior and fundamental performance of MNCP were also investigated. The results indicate that alkali cations on a montmorillonite layer surface were exchanged by using CTAB under 80 °C, successfully achieving the organic modification of montmorillonite. As a pore-forming agent, the modified montmorillonite caused a reduction in shrinkage: the 28-day autogenous shrinkage at a design density of 400 kg/m^3^ and 800 kg/m^3^ was reduced to 2.05 mm/m and 0.24 mm/m, and the highest reduction percentages during the 28-day drying shrinkage were 68.1% and 62.2%, respectively. The enlarged interlamellar pores and hydrophobic effects caused by the organic modification of montmorillonite aided this process. Organic-modified montmorillonite had a minor influence on dry density and thermal conductivity and could contribute to an enhancement of strength in MNCP.

## 1. Introduction

Cement-based porous materials are popular in building insulation due to their thermal insulation and energy-saving properties, light weight, simple processing, low cost, fire safety, and carbon sequestration [1,2,3]. However, compared to organic thermal insulation materials, their performance is significantly lacking [4,5,6]. To solve this problem, the thermal insulation performance of cement-based porous materials must be improved from the perspective of pore structure optimization [7]. However, the thermal conductivity of porous cement-based materials remains high due to the large thermal conductivity value of the cement-based matrix and the fact that the decreased percentage of thermal conductivity caused by pore structure optimization is highly limited [8,9]. Moreover, high porosity or low density can also be used to reduce thermal conductivity and improve thermal insulation ability [10,11], since the thermal conductivity of the increased phase (air) is extremely low. Usually, air voids, treated as macroscopic harmful pores, lead to a significant weakening of mechanical properties; therefore, achieving the balance of good thermal insulation performance and mechanical properties is difficult [12,13], greatly limiting the widespread application of cement-based porous materials.

To resolve this critical problem, the replacement of macroscopic pores (air voids) with microscopic pores was proposed to mitigate damage to the mechanical properties of pores and improve their thermal insulation performance [14]. This is because the decrease in pore size could cause a significant reduction in the thermal conductivity of the gas phase in pores and largely extend the heat transfer path of the solid phase [14,15]. Therefore, this approach is regarded as a promising method for balancing insulation performance and other properties, such as mechanical strength. Usually, macroscopic pores (air voids) can be replaced by microscopic pores using pore-forming media, such as aerogels [16,17], but the high cost of aerogels hinders their widespread application. Jiang et al. [14] developed low-cost, montmorillonite-based, pore-forming media which can also be used to construct rich microscopic pores in a cement matrix and was successfully utilized to prepare nanopore-rich cement pastes. However, due to the construction of rich nanopores, the significant shrinkage of these pastes often occurs, leading to a tremendous risk of cracking, which seriously affects their durability and applications. Significant shrinkage is currently the greatest challenge in this field of research.

Researchers have adopted many approaches to reducing the shrinkage of porous materials, such as adding various types of fibers, shrinkage-reducing agents, and expansion agents [18,19,20,21]. However, due to the light weight of porous materials, the contact surface between fibers and the cement matrix is minimal, and the shrinkage limitation ability of fibers is weakened. A shrinkage reducer is often used to reduce the shrinkage in concrete. For porous materials with a high number of microscopic pores, the critical dosage of the admixture for reducing shrinkage is relatively high, since the shrinkage reducer should be present in all microscopic pores and maintain a certain concentration. Expansion agents can also reduce shrinkage to a certain extent due to their expansion capacity, but a reduction in shrinkage may be difficult to guarantee during curing and serving processes, because water loss may occur at any time. Shrinkage cannot be effectively controlled using these external interventions [22,23]. Thus, shrinkage reduction should be carried out based on the source of shrinkage generation. Shrinkage stemming from water loss in small pores and the Young–Laplace equation indicate that small pores (≤10 nm) in cement-based materials generate great shrinkage stress when water migrates from these pores [24]. In particular, smaller pores cause greater shrinkage stress than larger pores [25]. For nanopore-rich cement-based materials prepared using a montmorillonite-based pore-forming agent, these extremely small nanopores (interlaminar pores) generate high shrinkage stress and are strongly related to the structure of montmorillonite. A montmorillonite unit contains two tetrahedral silica sheets and an octahedral alumina centrally located and sandwiched between two tetrahedral sheets. Usually, a montmorillonite layer has a negative charge due to the isomorphic substitution of Al^3+^ by Mg^2+^ in the octahedral sheet and Si^4+^ by Al^3+^ in the tetrahedral sheet [26]. This layer is often balanced, since it attracts alkali or alkaline earth cations at the mineral layer surface. To maintain this charge balance, these layers are rearranged into a multilayer structure: the cations interlaminate, stabilize the position of layer, and form rich and extremely small pores. However, the cations are easily exchanged for organic cationic surfactants, ultimately achieving the intercalation of organic cationic surfactants. This is often called the organic modification of montmorillonite [27,28]. After being intercalated, the chains of surfactants must extend to interlamination, due to space limitation, which increases the amount of available space, finally resulting in an enlargement of the interlayer pores [29,30]. Based on the shrinkage mechanism and the Young–Laplace equation [24], these enlarged pores tremendously reduce shrinkage stress, contributing to shrinkage reduction. Moreover, the carbon chain of surfactants often generates a hydrophobic effect. After organic modification, the carbon chain of surfactants can endow the interlamination with hydrophobic ability. Water cannot easily penetrate these interlayer spaces, helping to control shrinkage [31,32], since water loss is the precondition of shrinkage. Therefore, intercalation via organic modification may be effective in reducing shrinkage and preparing low-shrinkage nanopore-rich cement-based materials, demonstrating great potential in addressing the challenge of shrinkage control, which is seldom studied.

In this study, the organic modification of a montmorillonite-based pore-forming agent was conducted first, and then nanopore-rich cement pastes were prepared using the modified pore-forming agents. Their drying shrinkage and autogenous shrinkage were subsequently measured to detect their effectiveness in reducing shrinkage, and their hardened performance was investigated. The results in this study could inspire innovative approaches to effectively reducing shrinkage based on its source, contributing to lowering the risk of cracking, and thus guaranteeing the durability of nanopore-rich cement-based materials and promoting their applications in building insulation fields. The successful application of these high-performance, nanopore-rich, cement-based materials will narrow the gap between organic insulation materials and fire-safe, cement-based materials, greatly improving energy efficiency, ultimately saving energy usage in buildings.

## 2. Materials and Methods

### 2.1. Raw Materials

**Figure 1:**
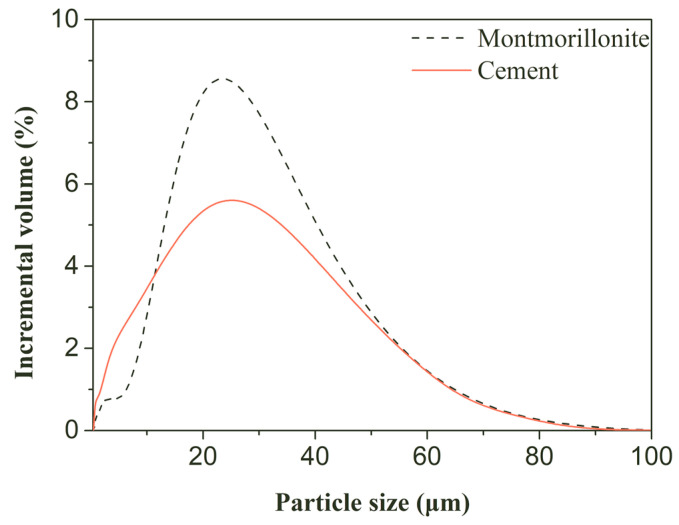
Particle size distribution of cement and montmorillonite.

Portland cement that met Chinese standard GB 175 [33] (P·O 42.5 R; initial setting time, 3.8 h; final setting time, 4.8 h; and 28-day compressive strength, 51.0 MPa; purchased from Lafarge Cement Plant in Jiangyou City, China) was used as a binder to prepare nanopore-rich cement pastes, and its particle size distribution is presented in Figure 1. Montmorillonite is a commercial product (bentonite) that was purchased from a Chinese company (Weifang Shengshi Co., Ltd., Weifang, China); its particle size distribution is shown in Figure 1. Montmorillonite contains quartz, feldspar, and illite. The modifier, composed of cetyltrimethyl ammonium bromide (CTAB), is of analytical grade and was provided by Fucheng Chemical Co., Ltd. (Tianjin, China).

### 2.2. Mix Design and Preparation

According to reference [34], a slurry of 18% montmorillonite can be used as nanopore-forming agent to fabricate nanopore-rich cement pastes. Before preparation, montmorillonite was mixed with water at 7000 r/min for 1 h; the concentration of montmorillonite was 18% by weight, and the slurry temperature was maintained at (20 ± 2) °C during the mixing process. Subsequently, montmorillonite slurry was placed in a room-temperature environment (temperature 20 ± 2 °C) for 24 h to fabricate an original nanopore-forming agent. For the facile organic modification of the nanopore-forming agent, various dosages of CTAB were added to the original montmorillonite slurry (concentration of 18%), and then this slurry was kept at 80 °C for 2 h under a mixing speed of 60 r/min to achieve organic modification. Following these procedures, the modified nanopore-forming agent was washed twice using deionized water to remove excess CTAB, and then organic montmorillonite (O-MMT) was obtained. However, when organic montmorillonite (O-MMT) was used as a nanopore-forming agent, the concentration of O-MMT slurry was adjusted to 18%.

O-MMT slurry and MMT slurry were used as nanopore-forming agents for the fabrication of MNCP. The content of the nanopore-forming agent was calculated based on design density, and the density of MNCP was strongly associated with the solid phases in the system, such as cement and MMT or O-MMT. A nanopore-forming agent that replaced cement paste in multiple experiments could be used to obtain a suitable design density due to its lower density compared to that of cement paste. According to this design philosophy and reference [34], two batches of MNCPs were designed, and the mix proportions are shown in Table 1.

Water was first added to a mixer, into which cement was poured and mixed with water (90 s) at 400 r/min to obtain cement paste. Subsequently, the nanopore-forming agent was introduced into the cement paste and mixed at the same speed until a homogenous MNCP slurry was obtained. Finally, this slurry was placed into molds, covered with plastic films, and cured at (20 ± 2) °C in a chamber of 90% relative humidity (RH) to achieve hardening. After hardening, these samples were unmolded, and some of them were covered with tinfoil for autogenous shrinkage test. Others were cured in the same environment in a hardened performance test, and a 28-day cured sample was used for the drying shrinkage test.

### 2.3. Test Methods

The hardened performance of MNCPs, which were fabricated using an unmodified or modified pore-forming agent, was evaluated using a strength test after 7-day, 28-day, and 56-day curing, based on ISO 679 [35]. Three samples of each mixture were used to obtain the strength at a specific age, and the loading rate for all samples was 2.4 kN/s. The equipment was a microcontrolled electronic universal testing machine (SANS, CMT5105, Shenzhen, China). Three 28-day-cured samples for each composition were used to obtain the thermal conductivity using the hot-desk method (DRE-2C, Xiangyi Instrument Co., Ltd., Xiangtan, China) in accordance with ISO 22007 [36]. Before the test, all samples were dried at 80 °C and polished.

For the dry shrinkage test, three 28-day-cured samples (40 mm × 40 mm × 160 mm) from each mixture were completely immersed into water at (20 ± 2) °C for 3 days. Then, these samples were taken out and free water at the surface was wiped out; the initial length was determined using dial gauges (BC 300, Tianjing Jianyi Instrument Co., Ltd., Cangzhou, China). Subsequently, the samples were placed in an environment of humidity (50 ± 5) % and temperature (20 ± 2) °C, and the mass and volume changes were tested according to Chinese standard JC/T 603 [37]. For autogenous shrinkage, an experiment was conducted based on ASTM C1698, and the volume change in the sample between the final setting and unmolded state was recorded using YC-BWS (Beijingyichuangshidai Technology Co., Ltd., Beijing, China). After the MNCPs (40 mm × 40 mm × 160 mm) were unmolded, they were covered with aluminum tinfoil and sealed using paraffin to prevent water loss, and then the length was continuously tested using the same dial gauges.

Based on the shrinkage-generated mechanism of cement-based materials, small pores exert a great influence on shrinkage, especially small nanopores, which dominate the shrinkage of cement-based materials [24]. Nitrogen adsorption/desorption isotherms can lead to these characteristics in the pores. More importantly, isotherms were obtained based on the absorption and desorption of nitrogen molecules, which are nondestructive. The isotherms were measured using an Autosorb-IQ (Quantachrome, Boynton Beach, FL, USA) at the temperature of liquid nitrogen in a range of relative pressures from 0.05 to 0.99. The measurements were conducted on samples in three replicates. After the isotherms were obtained, the pore size distributions were determined based on the level of the nitrogen adsorption and determined using the BJH method. However, before this test, these samples were dried at 60 °C in a vacuum-drying oven until their mass remained unchanged.

Montmorillonite were detected using an X-ray diffractometer (XRD, Cu target, D8 ADVANCE diffractometer, Brucker, Germany, 10°/min). Prior to this measurement, MMT or O-MMT slurry was dried in the same environment, as mentioned above, and then these dried materials were ground until all particles were smaller than 80 μm. Subsequently, these powders were loaded onto a plate sample holder via side loading to reduce preferred orientation effects, and then moved in the diffractometer. The powder diffraction curves were recorded using the equipment for further analysis. Moreover, these particles were used to obtain Fourier-transform infrared spectroscopy curves using a SPECTRUM ONE AUTOIMA (PerkinElmer, Waltham, MA, USA) in the range of 4000–400 cm^−1^ and at a resolution of 4 cm^−1^ to evaluate the modified results. The background material was FTIR-grade ground KBr.

## 3. Results and Discussion

### 3.1. Montmorillonite Modification

**Figure 2:**
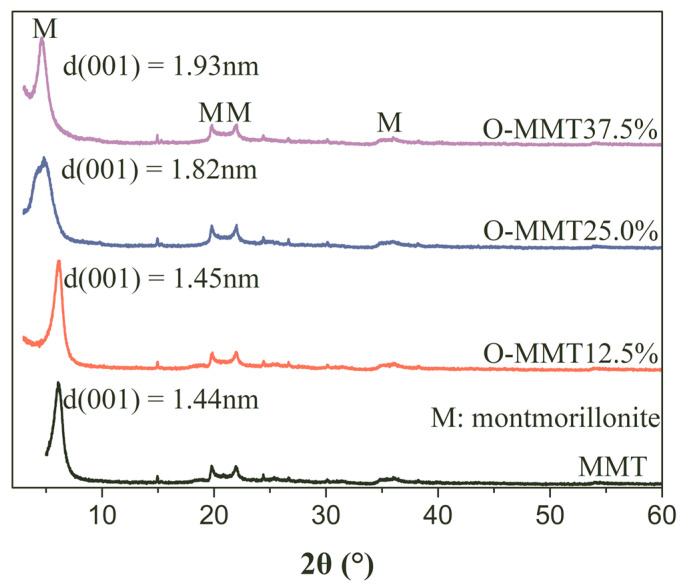
XRD patterns of montmorillonite (MMT) and organic montmorillonite (O-MMT).

Single montmorillonite (MMT) layers or tactoids can be used to separate and refine the capillary space of the cement matrix to generate nanopore spaces, and these nanolayers or tactoids can be fixed in the inner pores due to the interaction between the MMT layer and hydration products; therefore, these nanopore spaces could not be destroyed by conventional drying and formed rich nanopores. However, it was difficult to make all of the layers react with hydration products and generate enough hydration products at the MMT layers or tactoids. Therefore, these layers might be transformed into multilayers, forming part of the interlaminar pores, which are extremely small and have a major effect on increasing shrinkage [24]. The Young–Laplace equation indicates that the enlargement of these extremely small pores is effective in reducing shrinkage stress, and thus increasing the size of the original interlaminar pores of MMT might be a potential and effective approach to reducing their shrinkage values. The intercalation of montmorillonite is one of the most common ways of enlarging the interlayer pore size [38]. As shown in Figure 2, the main mineral phase was montmorillonite. The change in the 001 peak corresponding to the layer structure indicates that adding a modifier to the original nanopore-forming agent (MMT slurry) significantly increases the distance of interlayers, because the associated peak (001) of the MMT interlaminar pore moved in the direction of the small theta degree. Specifically, when the content of a modifier increases from 0 to 37.5%, the position of the 001 peak in MMT shifts from 6.1° to 4.7°, while the interlaminar distance of the MMT increases from 1.44 nm to 1.93 nm (an increase of 34.0%). When the content of modifier is 25%, the interlaminar pore size increases to 1.82 nm and by 0.11 nm. An excess modifier is not necessary, and the optimized dosage of the modifier is 25%.

**Figure 3:**
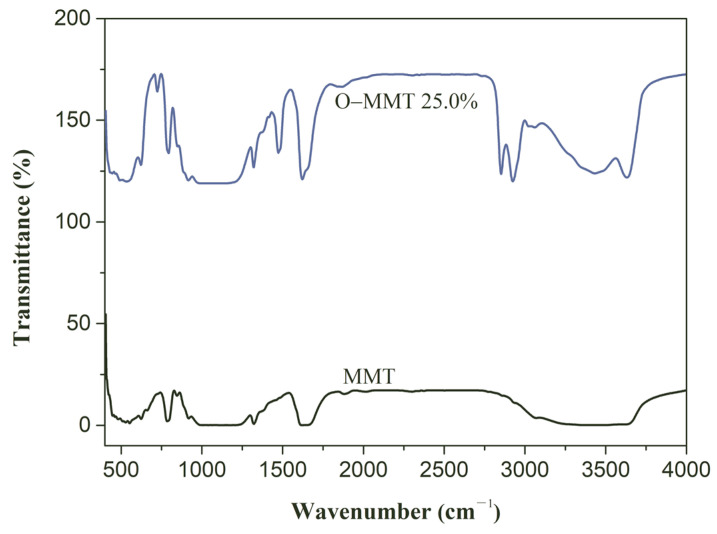
FT−IR patterns of montmorillonite (MMT) and organic montmorillonite (O-MMT).

**Figure 4:**
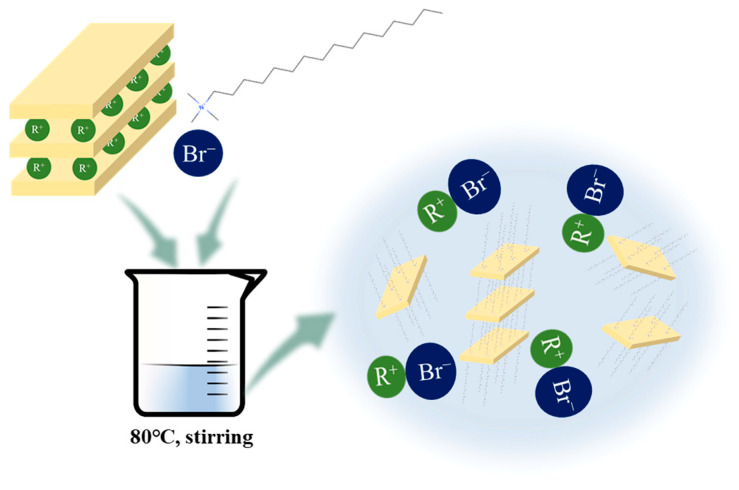
Schematic diagram of the facile modification of montmorillonite (MMT).

The modifying result is also reflected in the curves of the Fourier-transform infrared spectroscopy. As shown in Figure 3, the MMT has two distinct absorption bands at the high-frequency region. Bands of 3430 cm^−1^ and 1620 cm^−1^ correspond to the vibration of H-OH [39]. The band at 3628 cm^−1^ is associated with the functional group Al-OH [40]. The peak of Si-O-Si was observed at 790 cm^−1^ [40]. The weak absorption band at 912 cm^−1^ is related to the Al-OH in the vibration [41]. The bands at 445 cm^−1^ and 526 cm^−1^ might be coupling vibrations of OH and Si-OH [38,42]. In the O-MMT, the characteristic bands of the modifier occur. Due to multiple washing, the unreacted modifier can be removed. The characteristic bands, such as those of C-H at 2860 cm^−1^ and 2929 cm^−1^, are only from O-MMT [43]. This is attributed to the ions exchange between modifier and the MMT layer. A montmorillonite unit contains two tetrahedral silica sheets and an octahedral alumina centrally located and sandwiched between two tetrahedral sheets. Usually, a montmorillonite layer has a negative charge due to the isomorphic substitution of Al^3+^ by Mg^2+^ in the octahedral sheet and Si^4+^ by Al^3+^ in the tetrahedral sheet. This layer is often balanced by alkali or alkaline earth cations attracted to the mineral surface, and these cations are easily exchanged by organic cationic surfactants (such as CTAB); therefore, the chain of CTAB can be absorbed on the surface of the layer, as presented in Figure 4. The results of the XRD demonstrate the greater interlaminar pore size (Figure 2). Therefore, it is not difficult to infer that the absorbing site is on the layer surface and not at the end of the layer (Figure 4). This is because the interlaminar space is formed by two opposite layers and the enlarged interlaminar pore size only occurs under the conditions of an existing CTAB chain in the interlaminar space. This also reveals that the carbon chain radiated from the layer surface to the interlaminar space (Figure 4), since the distribution of this molecular chain can increase the layer space [43]. Therefore, with these facts in mind, the peaks of C-H at 2860 cm^−1^ and 2929 cm^−1^ indicate that the modifier successfully entered the interlaminar space; dried O-MMT has a larger interlaminar space than that of MMT [30].

**Figure 5:**
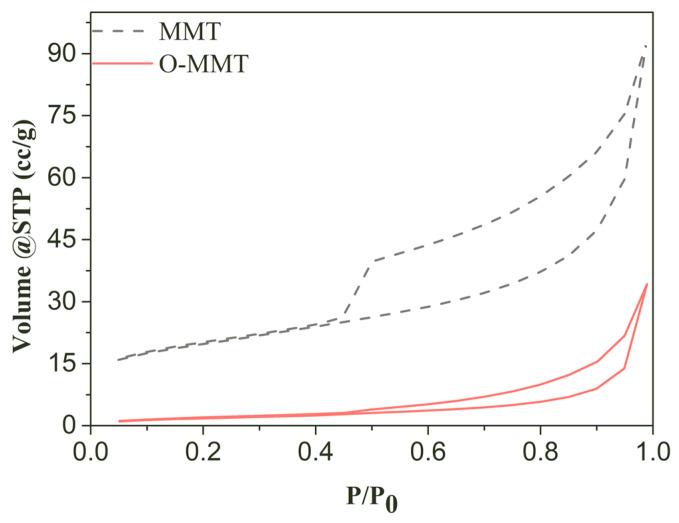
N_2_ adsorption/desorption isotherms of montmorillonite (MMT) and organic montmorillonite (O-MMT).

The interlaminar structure is characterized by nitrogen adsorption/desorption, as shown in Figure 5, indicating a larger hysteresis loop area and higher adsorption and desorption value. This demonstrated that the pores were formed by layer stacking and enlarged. With these facts in mind, the MMT was successfully modified by using CTAB under facile conditions (as mentioned in Section 2.2; the related mechanism is described in Figure 4). When the modifier was introduced into the MMT slurry under a facile modification environment, cation exchange in the MMT occurred. Alkali cations, which are attracted to the mineral surface, were exchanged by organic cationic surfactants, ultimately achieving the intercalation of organic cationic surfactants. Moreover, due to intercalation phenomena, the carbon chain of the modifier radiated from the layer surfaces to the interlaminar space, increasing the interlayer distance, which can enlarge the pore size [30]. More importantly, these chains near the layer surface improved the hydrophobic ability of the MMT layer, establishing that water could not easily penetrate the layer space, showing the hydrophobic effect of interlayer spaces, which prevented shrinkage due to water loss [31,32].

### 3.2. Drying and Autogenous Shrinkage

**Figure 6:**
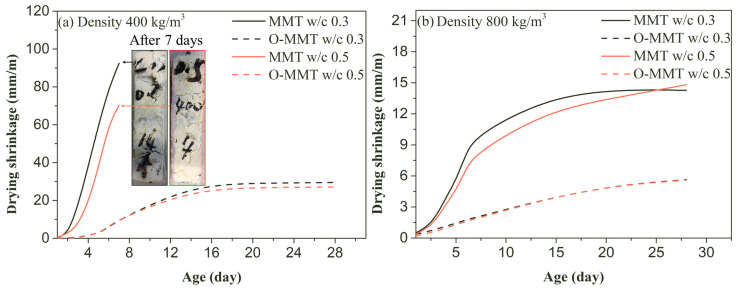

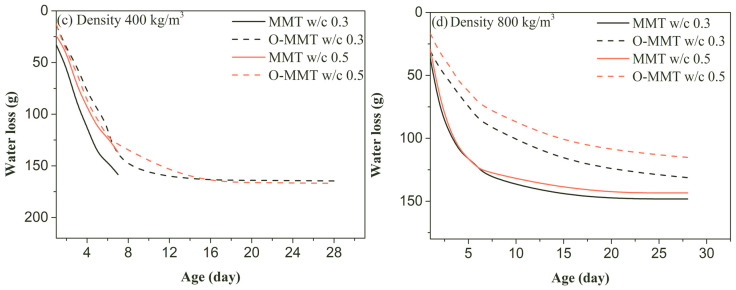
Drying shrinkage and water loss of montmorillonite-induced nanopore-rich cement paste (MNCP) at various ages.

As mentioned above, 25% of the modifier was suitable for fabricating O-MMT. After the modification of MMT, these modified MMT (O-MMT) and original MMT slurries were used as pore-forming agents to fabricate nanopore-rich cement pastes. The drying shrinkage of montmorillonite-induced nanopore-rich cement paste (MNCP) is presented in Figure 6. The shrinkage of low-density MNCP (design density 400 kg/m^3^) rapidly increased within 14 days, and the shrinkage changed slightly after 14-day exposure to a dry environment. The water loss in the low-density sample also experienced a similar phenomenon; the main water loss in the low-density sample was distributed across 14 days, and exposure for extended periods of time reduced the water loss rate. For the low-density samples, the shrinkage at the same exposure age was effectively limited by the complete replacement of the O-MMT nanopore-forming agent. For example, at 7 days of exposure, the shrinkage value of the low-density sample (MMT) was 92.50 mm/m (w/c = 0.3) and 70.33 mm/m (w/c = 0.5). Further extending the age of exposure resulted in the fragmentation of the unmodified samples (MMT). Therefore, the shrinkage values of these samples cannot be continuously measured, as shown in Figure 6. However, when a modified nanopore-forming agent (O-MMT) was used to prepare MNCPs, severe shrinkage and the broken phenomenon did not occur, and the 28-day shrinkage values of the low-density samples were 29.52 mm/m and 27.13 mm/m, respectively. For the w/c of 0.3 and 0.5, the reduction rates were high: 68.1% and 61.5%, respectively.

For the high-density samples (design density 800 kg/m^3^), the main water loss focused on the first 7 days. However, when the sample was continuously exposed to a dry environment, the water loss and shrinkage remained high because more complex and smaller pores in the high-density samples made water migration more difficult, and thus the significant loss of water took longer. Shrinkage is caused by water loss; therefore, the shrinkage of MNCP (high density) rapidly increased during the first 7 days of exposure, as shown in Figure 6, and extending the exposure time resulted in relatively lower shrinkage values. For the high-density and unmodified samples (MMT in Figure 6), severe shrinkage still occurred, but the broken phenomenon disappeared due to the lower content of pores and higher strength. Similar to the changing trend in the low-density sample, the shrinkage values of the modified sample fabricated by using the O-MMT slurry were lower than those of the unmodified sample (MMT). For instance, at 28 days of exposure, the shrinkage values of the high-density MMT sample were 14.50 mm/m and 14.81 mm/m, larger than those of the O-MMT sample (5.60 mm/m and 5.60 mm/m). The reduction rates of shrinkage were 61.4% and 62.2%, respectively, following the complete replacement of the O-MMT slurry, demonstrating a significant effect on drying shrinkage reduction.

**Figure 7:**
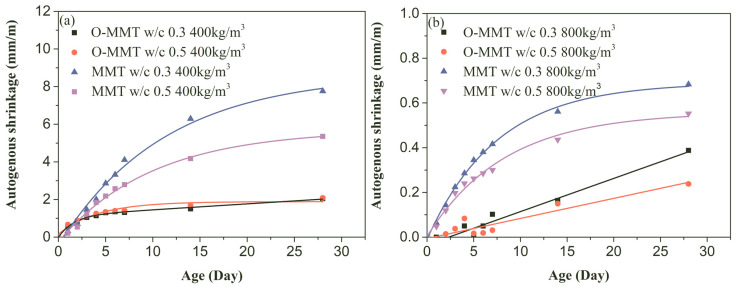
Autogenous shrinkage of montmorillonite-induced nanopore-rich cement paste (MNCP) at various ages. (**a**) Sample with design density of 400 kg/m^3^; (**b**) sample with design density of 800 kg/m^3^.

Self-desiccation happened during the cement hydration process, which caused a decrease in the internal relative humidity of the cementitious system and generated shrinkage; this shrinkage phenomenon is defined as autogenous shrinkage [44]. According to ASTM C1698 [45], the autogenous shrinkage value was recorded after the final setting of the cement slurry. Figure 7 shows the change in the autogenous shrinkage value of the MNCP at different curing ages. The autogenous shrinkage value of the sample rapidly increased within 14 days when an unmodified nanopore-forming agent (MMT) was used. However, for the fabrication of a low-density sample, the O-MMT slurry completely replaced the MMT slurry; the shrinkage value changed slightly after 3 days, and rapidly increased during the first 3 days. For the high-density sample, the autogenous shrinkage of the modified sample (O-MMT) continuously increased when the curing time changed from 0 to 28 days.

Autogenous shrinkage stemmed from water consumption (caused by cement hydration). When O-MMT is used for low-density MNCP, the dosage of O-MMT is large, playing a vital role in the change in shrinkage. This is because the hydrophobic effect of the carbon chain of the modifier (in O-MMT) means that water cannot be present in the interlamellar pore spaces [46]. However, the water in MMT is rich and sufficient time is required for its consumption, meaning that this process takes longer than the consumption of the modified sample (O-MMT), ultimately causing greater shrinkage. This shrinkage still occurred within 14 days for the MMT-fabricated sample and in just 3 days for O-MMT-fabricated sample.

For the high-density O-MMT sample, autogenous shrinkage was mainly controlled by cement hydration due to the high content of cement and low dosage of the nanopore-forming agent (Table 1), and the effect of the O-MMT (because of the little water in the interlamination due to the hydrophobicity of the modifier) was minor. Generally, it is impossible to complete cement hydration efficiently, and thus the autogenous shrinkage value increases over longer periods of time for the O-MMT sample. For the high-density MMT sample, the autogenous shrinkage caused by water consumption in interlamellar pores was large, and the consumption of the water generated the main shrinkage stress, and thus autogenous shrinkage from cement hydration could be ignored. Therefore, similar to autogenous shrinkage in the low-density sample (MMT), the continuous increasing phenomenon after 14 days was not significant, and autogenous shrinkage rapidly changed before 14 days and increased slightly after 14 days. This was mainly attributed to the shrinkage caused by water consumption, and the continuous consumption of water in MMT over short time periods was caused by the early hydration of cement. Little water remained in the MMT after longer time periods due to the high consumption of water early on.

As shown in Figure 7, when the O-MMT slurry was used for the fabrication of high-density MNCP, the autogenous shrinkage values at 28 days were 0.39 mm/m (w/c = 0.3, 800 kg/m^3^) and 0.24 mm/m (w/c = 0.5, 800 kg/m^3^), and the reduction rates compared with those of the unmodified MNCP were 42.6% (w/c = 0.3, 800 kg/m^3^) and 56.4% (w/c = 0.5, 800 kg/m^3^), respectively; therefore, the shrinkage reduction effect was excellent. At 7 days, the shrinkage values of the unmodified sample were 0.42 mm/m (w/c = 0.3) and 0.30 mm/m (w/c = 0.5), respectively. When the O-MMT slurry was used, the shrinkage values of the modified sample changed to 0.10 mm/m and 0.03 mm/m, and the reduced percentages were 76.2% and 90.0%, respectively. At 3 days, the autogenous shrinkage of the modified sample was close to 0, and the values of the unmodified sample were 0.22 mm/m (w/c = 0.3) and 0.20 mm/m (w/c = 0.5), respectively.

Similar to the phenomenon of the high-density sample, when the MMT slurry was used for the low-density MNCP preparation, the autogenous shrinkage values were high: 7.76 mm/m (w/c = 0.3, 400 kg/m^3^) and 5.35 mm/m (w/c = 0.5, 400 kg/m^3^), respectively. However, these values decreased with the use of the O-MMT slurry and were 2.05 mm/m (w/c = 0.3, 400 kg/m^3^) and 2.08 mm/m (w/c = 0.5, 400 kg/m^3^), with reductions of 73.6% and 61.1%, respectively. For the 7-day curing, the autogenous shrinkage values of the modified sample were 4.11 mm/m and 2.79 mm/m at a w/c of 0.3 and 0.5, respectively. These values reduced to 1.31 mm/m and 1.41 mm/m under the replacement of the O-MMT slurry, and the reduced percentages were 68.1% and 49.5%, respectively. For the unmodified samples at 3 days, the related values were 1.46 mm/m (w/c = 0.3) and 1.20 mm/m (w/c = 0.5), replacing the MMT slurry with the O-MMT slurry and causing a reduction in shrinkage, and they were reduced to 1.05 mm/m and 1.14 mm/m, respectively.

**Figure 8:**
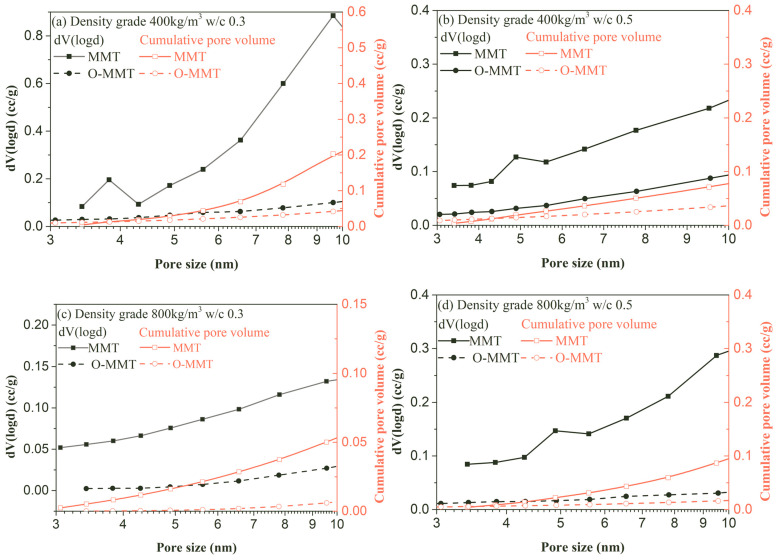
Pore size distribution of montmorillonite-induced nanopore-rich cement paste (MNCP).

As mentioned in reference [24], the reduction in shrinkage was also related to extremely small pores (pore size ≤ 10 nm) due to the extremely large shrinkage stress caused by the small nanopores and the low shrinkage stress caused by the large pores [24]. Thus, nitrogen adsorption/desorption was used to quantitatively characterize the pore structure of these small pores, as shown in Figure 8. When O-MMT was used to completely replace MMT, the cumulative volume and volume of the MNCP in the pores at the same w/c and density grade remarkably reduced. For the low-density samples, when the water-to-cement ratios were 0.3 and 0.5, respectively, the cumulative pore volume (≤10 nm) reduced from 0.21 cc/g to 0.06 cc/g and from 0.08 cc/g to 0.04 cc/g; the reduction percentages were high: 71.4% and 50.0%. For the high-density samples, the pore volume (≤10 nm) reduced from 0.06 cc/g to 0.01 cc/g and from 0.09 cc/g to 0.02 cc/g at a w/c of 0.3 and 0.5, and the reducing rates were 83.3% and 77.8%. Moreover, the pore volume at the same pore size generally decreased, as shown in Figure 8. The significant decrease in small pores contributed to the reduction in the shrinkage stress and had a major effect on shrinkage reduction in the MNCP. Moreover, water loss is the precondition of shrinkage. As mentioned in Section 3.1, the carbon chains radiated from the MMT layer surface to the outside, causing a hydrophobic effect and preventing water from approaching the MMT layer. Although MMT multilayers may form in the MNCP system, water cannot enter interlamellar spaces because these hydrophobic carbon chains exist in an interlamellar matrix. No water is present in this interlayer space, and thus, shrinkage stress from water loss can be significantly reduced, which contributed to reducing shrinkage in the MNCP. When O-MMT was used to replace MMT, it contributed to the fabrication of low-shrinkage MNCP as a kind of nanoporous material, and its shrinkage value was low [47,48,49]. Due to the reduced drying and autogenous shrinkage, the crack risk of the MNCP was greatly controlled, thus providing the possibility of applying cast-in-place or prefabricated MNCP in the external walls or roofs of buildings, which could further improve energy efficiency.

### 3.3. Fundamental Performance

**Figure 9:**
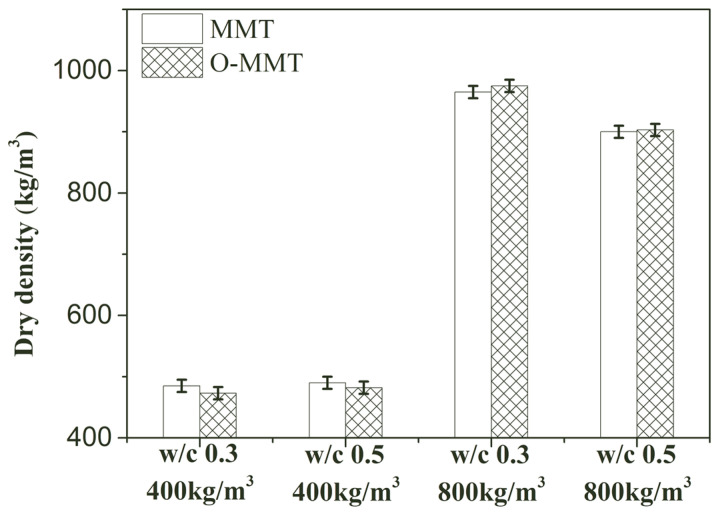
Dry density of montmorillonite-induced nanopore-rich cement paste (MNCP).

MMT modification was effective in controlling shrinkage, and the effect of this approach on the density, compressive strength, and thermal conductivity of the samples was also investigated. As shown in Figure 9, the dry density of the MNCP changed slightly. For the low-density grade of samples, the dry density of the MNCP changed from 485 kg/m^3^ to 473 kg/m^3^ and from 490 kg/m^3^ to 482 kg/m^3^ at a w/c of 0.3 and 0.5 when the O-MMT slurry was used to replace the MMT slurry. When the density grade of the sample was high, the dry densities of the sample varied from 965 kg/m^3^ to 975 kg/m^3^ and from 900 kg/m^3^ to 903 kg/m^3^ at a w/c of 0.3 and 0.5.

**Figure 10:**
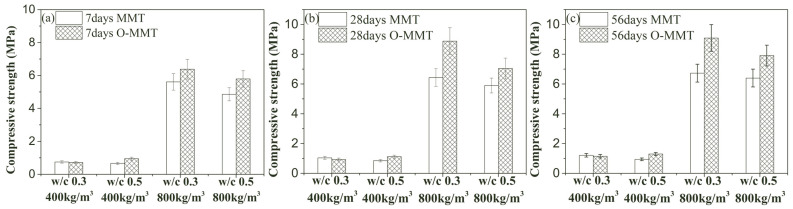
Compressive strength of montmorillonite-induced nanopore-rich cement paste (MNCP) at different ages: (**a**) 7-day compressive strength; (**b**) 28-day compressive strength; (**c**) 56-day compressive strength.

Due to the reduction in shrinkage stress, the generation of cracks was severely limited, thus reducing the primary cracks, which can improve the compressive strength of MNCP. As shown in Figure 10, the use of O-MMT for MMT replacement did not generate significant adverse effects on the strength of the sample at all ages and density grades. For example, when the dry density was low, the compressive strength at 7 days changed from 0.75 MPa to 0.72 MPa and from 0.66 MPa to 0.95 MPa at a w/c of 0.3 and 0.5, the related 28-day strength varied from 1.04 MPa to 0.93 MPa and from 0.85 MPa to 1.12 MPa, and the associated 56-day strength changed from 1.21 MPa to 1.15 MPa and from 0.94 MPa to 1.3 MPa, respectively. Similar to the phenomenon in the low-density sample, for the high-density sample, the 7-day, 28-day, and 56-day compressive strength values changed from 5.61 MPa to 6.38 MPa, from 6.43 MPa to 8.88 MPa, and from 6.73 MPa to 9.09 MPa at a w/c of 0.3; the percentage increases were 13.7%, 38.1%, and 35.1%, respectively. When the w/c was 0.5, the 7-day, 28-day, and 56-day compressive strength values increased from 4.86 MPa to 5.78 MPa, from 5.90 MPa to 7.04 MPa, and from 6.40 MPa to 7.91 MPa; the percentage increases were 18.9%, 19.3%, and 23.6%, respectively.

**Figure 11:**
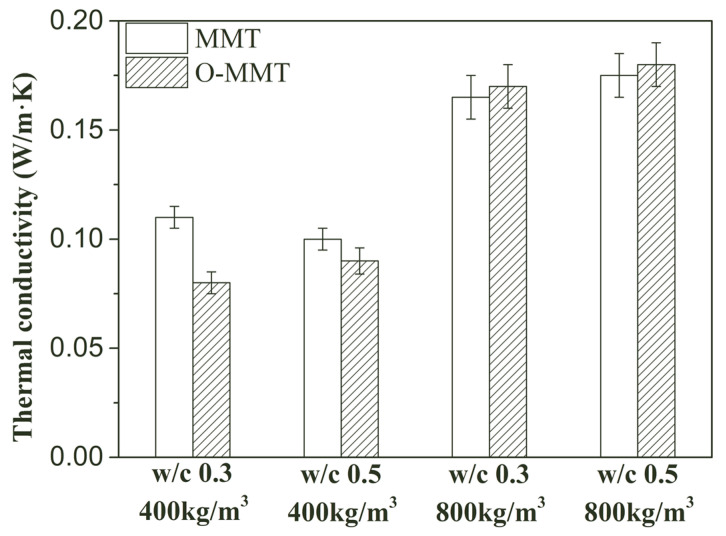
Thermal conductivity of montmorillonite-induced nanopore-rich cement paste (MNCP) at 28 days.

Shrinkage reduction contributed to controlling the cracks. When the density of the samples was low, cracks frequently occurred due to shrinkage. The cracks were often connective, which caused high heat convection and increased the thermal conductivity of the samples [50,51]. However, when a modified nanopore-forming agent (O-MMT) was used, the shrinkage value of the low-density sample significantly decreased, contributing to reducing cracks. A modified sample with a low density has a lower thermal conductivity compared to that of an unmodified sample, as shown in Figure 11. When MMT was completely replaced by O-MMT, the thermal conductivity reduced from 0.110 W/(m·K) to 0.080 W/(m·K) and from 0.100 W/(m·K) to 0.090 W/(m·K) at a w/c of 0.3 and 0.5; the reduction rates were 27.3% and 10.0%, respectively. For the high-density sample, the dosage of the nanopore-forming agent was low, as shown in Table 1. Shrinkage was relatively low and the skeleton of the pore was strong, demonstrating a strong ability to reduce cracks. Therefore, the connective cracks in the sample were scarce, ultimately causing a slight change in the thermal conductivity value, as shown in Figure 11 [13]. When the water-to-cement ratios were 0.3 and 0.5 for the high-density sample (design density: 800 kg/m^3^), the thermal conductivity values of the unmodified sample were 0.165 W/(m·K) and 0.175 W/(m·K), and the values of the modified sample were 0.170 W/(m·K) and 0.180 W/(m·K), respectively, showing a minor change. Combined with the positive effect of the modification on the strength and the minor effect on the thermal conductivity, MNCP can maintain good mechanical and thermal insulation performance. This means that it can be not only used in a sandwich structure as insulation materials, but can also be directly applied in an external building envelope as self-insulating wall or roof materials, improving the energy efficiency of buildings.

## 4. Conclusions

The organic modification of montmorillonite was successfully achieved by using cetyltrimethyl ammonium bromide under facile conditions, and montmorillonite-induced nanopore-rich cement paste (MNCP) was prepared to detect the effect of the organic modification of montmorillonite on the shrinkage behavior and fundamental performance. The main conclusions can be summarized as follows:

(1) Montmorillonite can be modified by using cetyltrimethyl ammonium bromide at 80 °C for 2 h, successfully achieving organic modification, which enlarges the interlayer pores and brings the hydrophobic chain into interlamination, hindering the penetration of water molecules.
(2) Autogenous and drying shrinkage were significantly reduced when organic-modified montmorillonite was used to replace original montmorillonite. The autogenous 28-day shrinkages at design density values of 400 kg/m^3^ and 800 kg/m^3^ were reduced to 2.05 mm/m and 0.24 mm/m, respectively, and the highest reduction percentages for the 28-day drying shrinkage were increased to at least 68.1% and 62.2%, respectively.
(3) Organic-modified montmorillonite has a minor influence on the dry density and thermal conductivity of MNCP, but it contributed to enhancing the strength of MNCP. The 56-day compressive strength of modified MNCP could be improved to 38.3% and 35.1% at densities of 400 kg/m^3^ and 800 kg/m^3^.
(4) The significant reduction in shrinkage, the enhancement of the mechanical performance, and the minor influence on the insulation performance showed that this process has great potential for applications in external building envelopes, and further research on the applications of nanopore-rich cement paste in building insulation will be necessary in the future.

## Figures and Tables

**Table 1:** Mix proportions of montmorillonite-induced nanopore-rich cement pastes (kg/m^3^).

Mix ID	Cement	Water	Water-to-Cement Ratio (W/C)	Nanopore-Forming Agent
MMT 400 0.3	212.1	63.9	0.3	909.6 (unmodified)
O-MMT 400 0.3	212.1	63.9	0.3	909.6 (modified)
MMT 400 0.5	219.1	109.6	0.5	856.6 (unmodified)
O-MMT 400 0.5	219.1	109.6	0.5	856.6 (modified)
MMT 800 0.3	577.5	173.2	0.3	671.0 (unmodified)
O-MMT 800 0.3	577.5	173.2	0.3	671.0 (modified)
MMT 800 0.5	596.4	298.2	0.5	527.1 (unmodified)
O-MMT 800 0.5	596.4	298.2	0.5	527.1 (modified)

## Data Availability

The raw/processed data required to reproduce these findings cannot be shared at this time, as the data also form part of an ongoing study.
